# The circulating transcriptome as a source of non-invasive cancer biomarkers: concepts and controversies of non-coding and coding RNA in body fluids

**DOI:** 10.1111/jcmm.12625

**Published:** 2015-06-27

**Authors:** Marta Fernandez-Mercado, Lorea Manterola, Erika Larrea, Ibai Goicoechea, María Arestin, María Armesto, David Otaegui, Charles H Lawrie

**Affiliations:** a Oncology Area, Biodonostia Research InstituteSan Sebastian, Spain; b Multiple Sclerosis Group, Biodonostia Research InstituteSan Sebastian, Spain; c Nuffield Division of Clinical Laboratory Sciences, University of OxfordOxford, UK; d IKERBASQUE, Basque Foundation for ScienceBilbao, Spain

**Keywords:** microRNA, ncRNA, biomarker, biological fluid, non-invasive

## Abstract

The gold standard for cancer diagnosis remains the histological examination of affected tissue, obtained either by surgical excision, or radiologically guided biopsy. Such procedures however are expensive, not without risk to the patient, and require consistent evaluation by expert pathologists. Consequently, the search for non-invasive tools for the diagnosis and management of cancer has led to great interest in the field of circulating nucleic acids in plasma and serum. An additional benefit of blood-based testing is the ability to carry out screening and repeat sampling on patients undergoing therapy, or monitoring disease progression allowing for the development of a personalized approach to cancer patient management. Despite having been discovered over 60 years ago, the clear clinical potential of circulating nucleic acids, with the notable exception of prenatal diagnostic testing, has yet to translate into the clinic. The recent discovery of non-coding (nc) RNA (in particular micro(mi)RNAs) in the blood has provided fresh impetuous for the field. In this review, we discuss the potential of the *circulating transcriptome* (coding and ncRNA), as novel cancer biomarkers, the controversy surrounding their origin and biology, and most importantly the hurdles that remain to be overcome if they are *really* to become part of future clinical practice.

- Introduction
- Circulating miRNAs as cancer biomarkers
  - Breast cancer
  - Prostate cancer
  - Colorectal cancer
  - Lung cancer
  - Haematological cancers
- Extracellular miRNAs in non-blood fluids
- Circulating mRNA
- Long non-coding (lnc)RNA and other ncRNAs
- Challenges in studying the circulating transcriptome
- Origin and function of cell-free nucleic acids
- Extracellular RNA of non-human origin: you are what you eat?
- Conclusion

## Introduction

Efficient management of cancer patients depends on early diagnosis and monitoring of treatment. The gold standard for cancer diagnosis remains the histological examination of affected tissue, obtained either by surgical excision, or radiologically guided biopsy. Such procedures however are expensive, not without risk to the patient, and require consistent evaluation by expert pathologists. Therefore, there has been great interest in the field of circulating nucleic acids as potential non-invasive cancer biomarkers 1,2. An additional benefit of blood-based testing is the ability to carry out screening and repeat sampling on patients undergoing therapy, or monitoring disease progression allowing for the development of a personalized approach to cancer patient management.

The first report of cell-free (cf) nucleic acids in biological fluids (blood) was made in 1947 by Mandel and Metais 3. However, with the exception of two reports on autoimmune diseases (systemic lupus erythematosus 4 and rheumatoid arthritis 5), the potential of circulating nucleic acids as biomarkers was not realized until 30 years later when Leon *et al*. reported high levels of circulating cfDNA in pancreatic cancer patients 6. Subsequently, in 1994, cancer-specific DNA mutations in *NRAS* (myelodysplastic syndrome 7) and *KRAS* (pancreatic cancer 8) were detected in the blood of cancer patients. In 1999, cfRNA was first detected in the blood of nasopharyngeal carcinoma patients 9, and in 2008, microRNAs (miRNAs) in the blood of diffuse large B-cell lymphoma (DLBCL) patients 10.

The discovery of circulating miRNAs in particular has led to a renewed interest in the field of circulating nucleic acids as biomarkers, and there are now more than 4500 publications on the subject. Below, we consider the potential of the circulating transcriptome (both coding and non-coding RNA) as a source of cancer biomarkers, their source and putative function along with some of the caveats that need to be considered when entering this rapidly emerging field.

## Circulating miRNAs as cancer biomarkers

The National Cancer Institute defines a biomarker as ‘a biological molecule found in blood, other body fluids or tissues that is a sign of a normal or abnormal process or of a condition or disease’. Cancer biomarkers are generally defined as being used for differential diagnosis (*diagnostic*), distinguishing between ‘good outcome tumours’ and ‘bad outcome tumours’ in the absence of treatment (*prognostic*) or assessing the probability that a patient will benefit from a particular treatment (*predictive*). A biomarker for clinical use ideally has high specificity, sensitivity and predictive power. Whether or not miRNAs will fulfil these criteria remains to be seen but they do have a number of characteristics (discussed below) that make them attractive candidates as biomarkers when compared to other classes of molecular biomarkers.

miRNAs are a recently discovered class of naturally occurring short non-coding (nc) RNA molecules that regulate eukaryotic gene expression post-transcriptionally. Over 2500 human microRNAs have been identified 11 and it is believed that more than 60% of all human genes are a direct target for miRNA regulation 12. miRNAs have been shown to play key regulatory roles in virtually every physiological and pathological aspect of biology 13, and there is now overwhelming evidence that dysfunctional expression of miRNAs is a ubiquitous feature of cancer 14,15.

Crucially for their role as potential biomarkers, differences in the expression profile of miRNAs can distinguish cancers according to diagnosis and developmental stage of the tumour to a greater degree of accuracy than traditional gene expression analysis, even discriminating between cancers that are poorly separated histologically 16. An especially useful characteristic of miRNAs as biomarkers is their remarkable stability which means not only can they be purified from routinely prepared formalin-fixed paraffin-embedded material 17 but they are also detectable in biological fluids 10. Unlike other RNA classes, the vast majority of which are degraded by high levels of RNases found in the blood 18, miRNAs appear stable in the blood and are surprisingly resistant to fragmentation by either chemical or enzymatic agents 19. Several studies using detergents, proteases and sonication suggest that miRNAs are not resistant to RNase degradation as a result of chemical modification but rather that they are protected by their lipid or protein carrier 19–21.

In 2007, we first reported the presence of miRNAs in the blood of cancer (lymphoma) patients 22 and in 2008, demonstrated their potential as cancer biomarkers 10. This was followed shortly after by Mitchell *et al*. who detected miRNAs in the plasma of prostate cancer patients 19. Subsequently, the field of circulating miRNAs has generated a great deal of interest and a multitude of publications expound the usefulness of this class of ncRNA as cancer biomarkers. Some of the major findings in this field are described below and listed in Table 1. In addition to cancer, circulating miRNAs also have great potential as biomarkers for many other diseases or medical conditions, including cardiac injury, autoimmune diseases, diabetes and toxicity, as well as their use in prenatal screening (reviewed elsewhere 23).

**Table 1:** Examples of deregulated levels of circulating ncRNAs in various malignancies in relationship with their diagnostic and prognostic value

Cancer type	Circulating ncRNAs	Clinical value	Body fluid type	Cohort size		*P*-value	Reference
				Patients	Controls		
Breast cancer	*let-7a, miR-195*	D	Serum	148	44	<0.001	31
	*let-7a, miR-195*	D	Blood	83	63	<0.001	161
	*miR-10b* *miR-34a* *miR-155*	D, P	Serum	89	29	0.005 0.001 0.0001	28
	*miR-21* *miR-146a*	D	Plasma	14	8	<0.0004 <0.005	162
	*miR-148b,* *miR-652, miR-801* *miR-127-3p* *miR-409-3p*	D	Plasma	277	140	<0.0001 <0.0001 0.0003 0.005	163
	*miR-125b*	PR	Serum	56	10	0.008	164
	*miR-122, miR-375*	P	Serum	68	–	<0.005	27
	*miR-155*	D	Serum	13	8	0.016	165
	*miR-182*	D	Serum	46	58	<0.01	166
	*miR-215* *miR-299-5p* *miR-411* *miR-452*	D	Serum	75	20	0.094 0.019 0.082 0.002	167
Prostate cancer	*let-7a, miR-145, miR-155*	D	Blood	20	63	≤0.001	161
	*let-7c, let-7e,* *miR-30c, miR-622* *miR-1285*	D	Serum	105	115	<0.001	168
	*let-7i* *miR-16* *miR-195*	D	Serum	73	20	0.022 0.023 0.05	36
	*miR-141, 16, 92a, 92b, 103, 107, 197, 34b, 328, 485-3p, 486-5p, 574-3p, 636, 640, 766 and 885-5p*	D	Serum	6	15	NA	127
	*miR-221, miR-21* *miR-20a* *miR-21* *miR-145*	D	Plasma	82	–	0.002 0.03 0.047 0.011	169
	*miR-107* *miR-574-3p*	D	Urine	78	28	<0.01	37
	*miR-375* *miR-141*	D	Plasma-derived microvesicles	78	28	<0.05	37
	*miR-141*	D	Serum	25	25	<0.001	19
	*miR-141* *miR-375*	P	Serum	116	–	<0.05 <0.01	39
	*lncRNA MALAT-1*	D	Plasma	217	–	<0.001	98
	*lncRNA PCA3*	D	Urine	517	–	NA	71
Colon cancer	*let-7a, miR-155* *miR-145* *miR-10b*	D	Blood	30	63	<0.001 0.001 ≤0.005	161
	*miR-17-3p, miR-92*	D	Plasma	90	90	<0.0005	42
	*miR-29a* *miR-92a*	D	Plasma	157	59	<0.0001	43
	*miR-29c*	P	Serum	61	23	0.012	45
	*miR-141*	P	Plasma	185	76	<0.005	40
	*miR-221*	D P	Plasma	103	37	0.0021 <0.05	44
	*RNU2-1f*	D	Blood	232	129	<0.05	170
Gastric cancer	*let-7a* *miR-17-5p* *miR-21* *miR-106a* *miR-106b*	D	Plasma	69	30	0.002 0.05 0.006 0.008 <0.001	171
	*miR-21*	P	Plasma	69	–	0.0133	172
	*miR-199a-3p*	D	Plasma	80	70	0.012	173
Oral cancer	*let-7b, miR-16, miR-29a, miR-223, miR-338-3p*	D	Serum	30	26	<0.05	174
	*miR-31*	D	Plasma Saliva	43 (plasma) 8 (saliva)	21 (plasma)	<0.0001	175
	*miR-125a* *miR-200a*	D	Saliva	50	62	<0.05	64
Ovarian cancer	*let-7f* *miR-205*	D	Plasma	360	200	0.008 <0.001	176
	*let-7f*	P	Plasma	360	200	0.006	176
	*miR-21, miR-29a, miR-92, miR-93, miR-126, miR-155, miR-127, miR-99b*	D	Serum	28	15	<0.01	177
	*miR-21, 141,200a, 200b. 203, 205, 200c, 214*	D	Serum-derived exosomes	50	20	≤0.05	178
	*miR-200a* *miR-200b* *miR-200c*	D	Serum	28	28	<0.05 0.05 0.0005	179
	*RNU2-1f*	D, PR	Serum	124	40	<0.0001 0.0015	104
Hepatocellular cancer	*miR-1* *miR-122*	P, PR	Serum	195	54 (cirrhosis)	0.011 0.036	180
	*miR-500*	D	Serum	40	–	NA	181
	*lncRNA HULC*	D	Blood	4	19	NA	182
Lung cancer	*miR-1,30d,486, 499*	P	Serum	303	–	<0.001	180
	*miR-10b* *miR-141* *miR-155*	D	Serum	35	35	0.002 0.0001 0.007	29
	*miR-25, miR-223*	D	Serum	152	75	<0.001	20
	*miR-375*	P	Plasma	217	217	<0.05	183
	*miR-125a-5p, miR-146a, miR-145*	D	Serum	70	70	<0.0001	184
	*miR-653, miR-660* *Cyfra21-1*	D	Serum	222	144	<0.01	47
	*miR-125-5p* *miR-25* *miR-126*	D	Serum	24	24	<0.001	185
Squamous cell carcinoma	*miR-18a*	D	Serum	106	54	<0.0001	186
	*miR-184*	D	Serum	30	38	0.002	187
B cell lymphoma	*miR-21* *miR-155* *miR-210*	D	Serum	60	43	0.04 0.009 0.02	10
	*miR-155*	Predict	Plasma	228	–	0.0303	188
Glioblastoma	*miR-21*	P	Serum exosomes	25	30	0.03	146
	*miR-21*	D	Plasma	10	10	0.02	189
Pancreatic cancer	*miR-21* *miR-210* *miR-155* *miR-196a*	D	Plasma	49	36	0.007 0.003 0.042 0.009	190
	*miR-210*	D	Plasma	22	25	<0.0004	191
	*miR-642b* *miR-885-5* *miR-22*	D	Blood	19	33	<0.001	192
	*RNU2-1f*	D	Blood	232	129	<0.05	170
Leukaemia	*miR-92a*	D	Plasma	61	16	<0.001	53
	*miR-150, miR-342*	D	Plasma	40	20	<0.01	56
Bladder cancer	*miR-126: miR-152* *miR-182: miR-152*	D	Urine	47	36	<0.01 <0.005	193
Rhabdomyosarcoma	*miR-206*	D	Serum	31	17	<0.001	194
Pleural mesothelioma	*miR-625-3p*	D	Plasma/Serum	45	24	0.004	195

lncRNA: long non-coding RNA; miR: microRNA; ncRNA: non-coding RNA; RNU: small nuclear RNA; D: diagnostic; P: prognostic; PR: Predictive of response.

In the interests of space, below we discuss some of the most important studies relating to only the most common forms of cancers (further examples can be found in Table 1). This section not intended to represent an exhaustive list of studies on the circulating miRNome, but rather to illustrate the weight of evidence that now exists suggesting that miRNAs great potential as novel non-invasive cancer biomarkers.

### Breast cancer

Expression levels of *miR-21*, *miR-126*, *miR-155, miR-199a* and *miR-335* in sera have all been associated with clinicopathological features of breast cancer, including histological tumour grade and receptor status 24. Circulating levels of *miR-214* were suggested to have diagnostic potential in breast cancer patients 25, and levels of circulating *miR-21* may have utility in detecting progression of early stage breast cancer 26. In another study, circulating blood levels of *miR-122*, *miR-10b*, *miR-34a* and *miR-155* were associated with the presence of overt metastasis 27,28. Interestingly, serum concentrations of the same miRNAs are also significantly elevated in the sera of patients with ovarian and lung cancer 29,30. Heneghan *et al*. found an association between high serum levels of *miR-10b* and the oestrogen receptor status of breast cancer patients 31. Additionally, it has been suggested that plasma *miR-210* levels could be used for monitoring the response of breast cancer patients to trastuzumab 32.

### Prostate cancer

A recent study demonstrated that a blood test based upon a combination of the levels of five circulating miRNAs (*let-7e, let-7c, miR-30c, miR-622 and miR-1285*) could effectively differentiate between prostate cancer and benign prostatic hyperplasia, as well as healthy controls 33. Plasma levels of *miR-20a, miR-21, miR-145* and *miR-221* were also suggested to be useful in distinguishing between prostate cancer patients of varying aggressiveness of tumour 34. *miR-125b* and *miR-141* have also been shown to be up-regulated in the sera of prostate cancer patients with metastasis in comparison to those of healthy controls 35. Similarly, elevated levels of *miR-16*, *miR-195* and *miR-let-7i* have been detected in prostate cancer patients’ sera 36. *miR-107* and *miR-574-3p* were also present at high concentrations, this time in the urine of prostate cancer patients 37. Patients with hormone-refractory prostate cancer expressed higher serum levels of *miR-21* than those with androgen-dependent and localized prostate cancer 38. Perhaps, most promising of the studies is the consistent finding that plasma *miR-141* has diagnostic potential for prostate cancer 19,37,39.

### Colorectal cancer

In addition to prostate cancer, high levels of plasma *miR-141* have also been associated with the presence of distant metastasis and poor prognosis in colorectal cancer 40. *miR-29a* has been proposed as a potential non-invasive biomarker for early detection of colorectal cancer involving liver metastasis 41 and, *miR-92* levels in plasma have been shown to be able to differentiate between colorectal cancer and gastric cancer patients, potentially conflicting diagnoses 42. In three consistent studies, *miR-29a, miR-92* and *miR-221* in plasma have been identified as potential biomarkers of colorectal cancer 42–44. In addition, the closely related sequence *miR-29c* was observed to be significantly increased in early relapsed patients compared to non-early relapsed patients 45.

### Lung cancer

Four miRNAs (*miR-486*, *miR-30d*, *miR-1* and *miR-499*) were identified in the serum of non-small cell lung cancer (NSCLC) patients that were linked to overall survival 46. In addition, in patients with lung carcinoma and lymph node metastases, particularly high concentrations of *miR-155, miR-141* and *miR-10b* were associated with disease, and the latter miRNA, with high concentrations of the tumour marker TPA 29. In a recent report, sera levels of *miR-652 and miR-660* were used in conjunction with the existing clinical biomarker Cyfra21 to improve the diagnostic power of adenocarcinoma NSCLC cases 47,48.

### Haematological cancers

Originally proposed in 2008 10, both *miR-21* and *miR-92* have been validated independently as potentially useful blood biomarkers of DLBCL 49,50. In addition, *miR-92a* has been proposed as diagnostic/prognostic biomarkers for multiple myeloma (MM) and acute leukaemia 51–53. In Hodgkin lymphoma, *miR-494* and *miR-1973* were identified indicators of both relapse and interim therapy response 54. Plasma *miR-221* has been found to be a good diagnostic and prognostic marker for extranodal natural killer/T-cell (NK/T-cell) lymphoma 55. *miR-150* and *miR-342* were shown to be promising biomarkers in the diagnosis of acute myeloid leukaemia (AML) 56, and *miR-181b-5p* has been suggested to be a good predictor for overall survival in AML patients 57.

## Extracellular miRNAs in non-blood fluids

**Figure 1:**
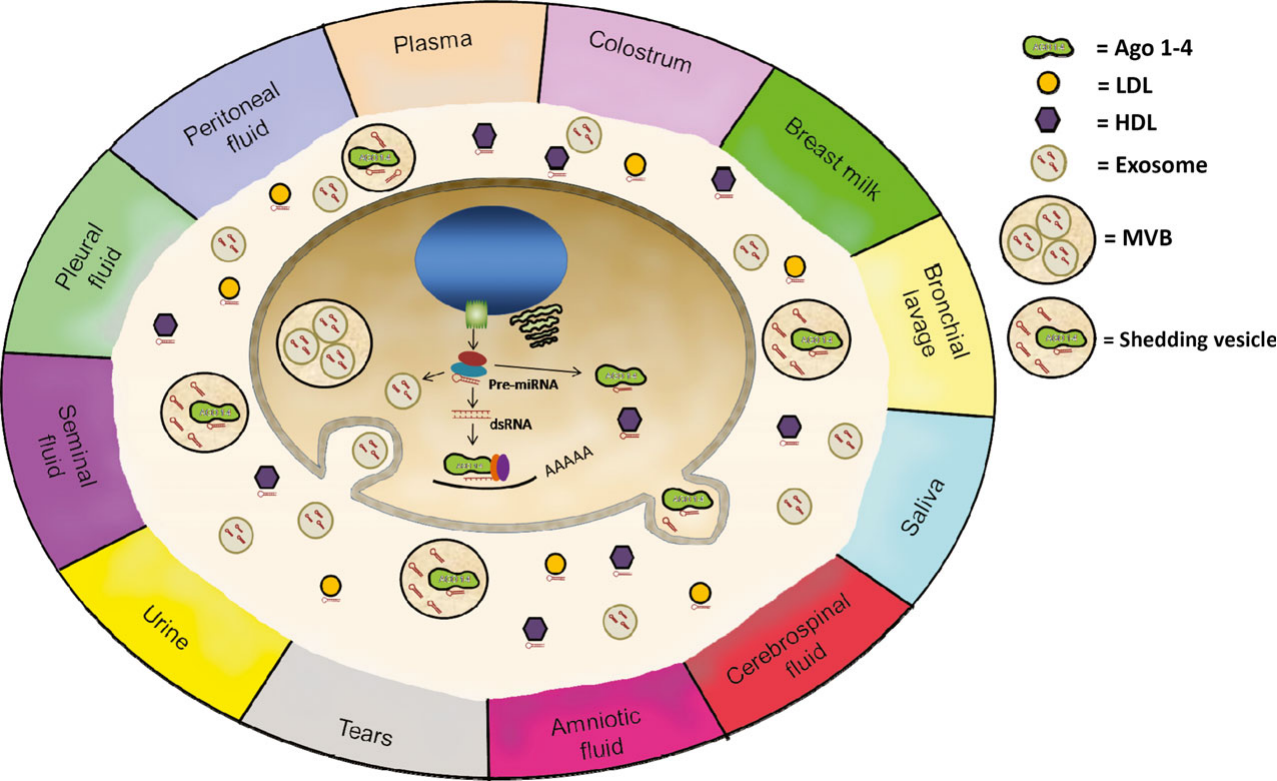
Origin of extracellular RNA. Several hypotheses have been proposed to explain the source of circulating RNA, including the passive release of RNA from broken cells and tissues following tissue injury, chronic inflammation, cell apoptosis or necrosis or from cells with a short half-life. Alternatively, active secretion of cfRNA can occur in association with subcellular components including exosomes, microparticles, microvesicles or extracellular vesicles 19,135–137. Additionally, there is emerging evidence for active secretion by cells as RNA-binding-protein conjugated complexes. Cell-free miRNA have been detected in 12 different body fluids: plasma, saliva, tears, urine, amniotic fluid, colostrum, breast milk, bronchial lavage, cerebrospinal fluid, peritoneal fluid, pleural fluid and seminal fluid 59. Ago 1–4: argonaute proteins 1–4; LDL: low-density lipoprotein; HDL: high-density lipoprotein; MVB: multivesicular body.

Although the vast majority of studies to date have been carried out in the blood, extracellular miRNAs are also present in many other biological fluids including saliva, tears, seminal fluid, breast milk, vitreous and aqueous humours of the eye and cerebrospinal fluid 58,59 (Fig. 1). These fluids appear to be particularly useful as biomarkers for cancers associated with their origin. For example, salivary RNA has been proposed as a useful biomarker for oral 60, head and neck squamous cell carcinoma 61, oesophageal cancer 62 and parotid gland tumours 63–65. In addition to miRNAs, mRNA (*IL-8, IL-1*) 66–68 and lncRNA have also been identified as potential saliva biomarkers for these cancers 69. Many studies have examined the potential of urine as a source of RNA biomarkers for urological cancers (reviewed in 70). Particularly promising in prostate cancer are *miR-107*, *miR-574-3p* and *PCA*, all of which have been described in multiple studies as diagnostic indicators of the disease 37,71. Apart from whole urine, the cellular component is a frequent source of miRNAs in studies, although sometimes cf urine or purified exosome preparations are also used 37,72–76. Several studies have examined the potential role of cerebral spinal fluid miRNAs in brain tumours 77,78, and recently milk has been identified as a potential supply of RNA biomarkers in breast cancer patients 79. In addition, the aqueous humour of eyes appears to be a promising source of extracellular miRNAs for diagnosing glaucoma 80. The potential of these non-blood biofluids as a source of biomarkers for tumours arising in non-associated sites remains unexplored.

## Circulating mRNA

Unlike miRNAs, the vast majority of extracellular mRNA in the blood is degraded by RNase activity and detectable fragments are typically less than 100 bp in length 81. There are however notable exceptions and some genes appear not to be degraded 82, presumably as a result of complexing with protein and/or lipid carriers. In 1999, Lo *et al*. first reported the presence of cfRNA in the plasma of nasopharyngeal carcinoma patients 9, and shortly after this was followed by the observation that cfmRNA was detectable in the serum of melanoma patients 83. In breast cancer, the presence of *cyclin D1* mRNA in plasma has been found to be associated with patients that were refractory to tamoxifen treatment and those that had a poorer clinical outcome 84, while increased *Bmi-1* mRNA levels were also found to correlate with poor clinical performance 85. In prostate cancer, *hTERT* mRNA has been linked with poor prognosis 86, and levels of *cBMP6* mRNA with metastatic disease 87. Many plasma mRNAs have been proposed as disease biomarkers for hepatocellular carcinoma, including *LMNB1, TGF*β and *MCM6* 88–90. In B-cell lymphoma, the presence of serum *MYC* mRNA is associated with short overall and progression-free survival as well as partial treatment response 91. Interestingly, despite the relatively long history of circulating mRNA discovery, this field has not translated into clinical practice, or captured the imagination of the scientific community in the same way as miRNAs, perhaps because of the lability and inter-individual variability in mRNA levels in the blood 92.

## Long non-coding (lnc)RNA and other ncRNAs

In addition to miRNA, many other classes of ncRNA are transcribed from the human genome. Indeed even though ∼75% of the genome is transcribed, miRNAs account for less than 2% of this output 159. While it is unlikely that all of the remaining ncRNA is functional, there is now significant evidence that ncRNA other than miRNA is essential for both physiological function and development, as well as playing a fundamental role in disease 93,94. Compared to miRNAs, however, there is very little research on these ncRNAs, although a number of different classes are now recognized. As functional information on all but a very few remains unknown, these classifications are based primarily on size and include short ncRNAs such as miRNAs, piRNAs and tiRNAs; mid-size ncRNAs such as snoRNAs, snRNAs, PASRs, TSSa-RNAs and PROMPTs; and long ncRNAs (lncRNAs) 94,95.

Compared with the 2000 or so human miRNAs, over 210,000 different species of lncRNA have been identified 96; yet very few studies have been carried out on this class of ncRNA. A notable exception is Prostate cancer antigen 3 (PCA3) in the urine of prostate cancer patients which has been intensively investigated and is potentially more specific than Prostate-specific antigen (PSA) levels (reviewed in 97). Levels of blood MALAT1 have also been proposed as a biomarker for this cancer 98, although in far fewer patients. A study comparing plasma from patients with chronic lymphocytic leukaemia and MM patients with healthy individuals found differing levels of five lncRNAs (TUG1, LincRNA-p21, MALAT1, HOTAIR and GAS5) 99. Higher levels of HULC lncRNA was observed in the plasma of patients with hepatocellular carcinoma than healthy individuals 100. Six lncRNAs were found to differ in the saliva of patients’ oral squamous cell carcinoma compared to controls and to have potential in identifying metastatic patients 69. Mitochondrial-derived lncRNA have also been proposed as biomarkers in the urine of bladder cancer patients 101. Levels of H19 lncRNA in the plasma of gastric cancer patients was found to be significantly raised when compared to healthy controls 102. Outside of cancer, circulating levels of LIPCAR lncRNA were found to predict survival in heart failure patients 103. Besides lncRNAs, levels of the small nuclear RNA (snRNA) U2 was increased in the blood of patients with ovarian cancer as well as being linked with the responsive to chemotherapy 104, and six small nucleolar RNAs (snoRNAs) were up-regulated in the plasma of NSCLC 105. To date, we are not aware of any reports on piRNAs or other forms of ncRNA in biological fluids.

## Challenges in studying the circulating transcriptome

The circulating transcriptome biomarker studies listed above (and in Table 1) are by no means an exhaustive list, but instead intended to illustrate the rapid growth of studies in this area. It should be noted, however, that the vast majority of research in this area are single-centred retrospective studies and that the cohorts typically used are insufficiently powered (Table 1). As a consequence, there are many non-overlapping and even contradictory reports relating to the circulating transcriptome. These differences are primarily because of biological and technical variation between studies such as the starting material used in experiments (*e.g*. purification of cells, cell types, control populations used, RNA extraction, *etc*.), technological platforms [*e.g*. microarray, qRT-PCR, *versus* next generation sequencing (NGS), *etc*.], and differing statistical methodologies used. Such confounding factors are especially problematic for studies of the circulating transcriptome which are characterized by low-quality and low-quantity RNA. Below, we discuss some of these issues in more detail.

Although obvious, the choice of starting material is crucial to initial experimental design and the choice of whole blood, peripheral blood mononuclear cells, serum, plasma or purified exosomes from the same individual will generate very different expression profiles 106–108. The first critical step in blood-based studies is collection and handling procedures. The receptacle used to collect the blood is crucial and should be ethylenediaminetetraacetic acid or citrate-containing, as heparin, a commonly used anticoagulant can inhibit the reverse transcriptase and polymerase enzymes used in PCR 109–111. The blood collection protocol is also vital, and should be optimized to reduce the time taken between phlebotomy and processing, and to avoid haemolysis which can be a major cause of variation in RNA levels not related to any biological difference 112–114. The choice of serum or plasma is also crucial to the experimental outcome as although some studies found no significant differences between serum and plasma levels of miRNAs 19,115, others observed that serum samples contain lower miRNA concentrations than plasma samples 106. In addition to the technical variables already mentioned, it is also important to bear in mind the advantages and disadvantages of choosing a particular sample because of their inherent characteristics, which may affect the performance of the downstream applications. A number of these features are listed in Table 2 for blood and urine collection.

**Table 2:** Summary of advantages and limitations of measuring RNA in the most commonly used biological fluids for biomarker discovery

	Plasma	Serum	Urine
Accessibility	Minimally invasive	Minimally invasive	Non-invasive
Applications	Any type of cancer	Any type of cancer	Renal, prostate and bladder cancer
miRNA stability	Stable under harsh conditions including boiling, low/high pH, extended storage and multiple freeze–thaw cycles 19,191	Stable under harsh conditions including boiling, low/high pH, extended storage and multiple freeze–thaw cycles 20,196	Stable under multiple freeze–thaw cycles 197
RNA quantity	10–300 ng/ml 59	10–300 ng/ml Conflicting reports: some report lower RNA yield than plasma 106, whereas others report similar yield 115	1–100 ng/ml 59,198
	miRNA levels strongly correlate between plasma and serum 19	miRNA levels strongly correlate between plasma and serum 19	
RNA quality	Degraded <1000 bp RINs >6 199,200	Degraded	Degraded
PCR inhibitors	Anticoagulants: heparin, citrate		
Interferences with extraction	High protein abundance	High protein abundance	
Cellular contamination	Haemolysis (control: miR-23a and miR-451)	Haemolysis (control: miR-23a and miR-451)	
	Blood cells no separated properly, Cell debris, apoptotic bodies, blood platelets	Cell debris, apoptotic bodies, blood platelets	Urethral cells, cell debris
	Frequent 114		

RNA: ribonucleic acid; RIN: RNA integrity number.

Another important source of variability comes from the choice of RNA purification procedure. On a cautionary note, Trizol-based extraction methods which are among the most common protocols used, could give biased results as low GC-content RNA can be lost during purification of biological fluids and other samples with low concentration of RNA 116. Furthermore, biological fluids typically contain very high levels of salts, lipids and proteins that can inhibit enzymes used to detect RNA. Many protocols use *Caenorhabditis elegans* (or other non-human) miRNAs added to the plasma sample as a spike-in to control for this (and extraction) variability 19. An additional issue is that because of the low quantities of RNA present in biological fluids, it is often impossible to measure RNA accurately, therefore studies often use fixed volumes of starting material that invariably contain differing RNA levels 92.

**Figure 2:**
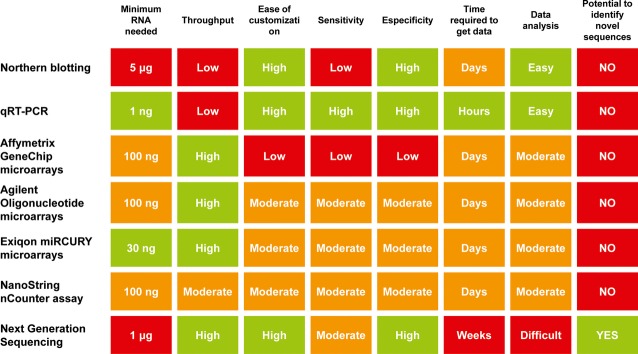
Comparison of methods commonly used to study extracellular RNA. Colour code indicates the relative feasibility of that particular technique based on a given feature, from green (more feasible), through orange, to red (less feasible). Data analysis: Easy (feasible in any molecular biology lab), Moderate (various software platforms available), Difficult (requires advanced computational infrastructure). Modified from Moldovan *et al*., 2014 119.

Many methods are routinely employed to measure extracellular RNAs including qRT-PCR (LNA-based, Taqman or other proprietary technologies), microarrays and more frequently NGS techniques. Each of these techniques has advantages and disadvantages depending upon the experimental design (Fig. 2). Several excellent publications have recently reviewed the technological issues associated to the different techniques in depth 117–119. It is not the aim of this review to recapitulate all these issues in detail; however, it should be noted that the choice of platform greatly influences the end result and a several reports have shown disparate results from the same sample source using different platforms 120,121.

Circulating transcriptome studies are confounded further by the lack of a standard approach to normalization or indeed a suitable endogenous reference gene. Although global mean normalization is probably the most accurate method for normalization when considering profiling studies, the low number of miRNA species (typically >100) present in biological fluids makes it unsuitable 122. Furthermore, even though snRNAs such as U6 or U48 are widely accepted as endogenous controls for miRNA cell-based studies, they are not present at detectable levels in biological fluids 123–125. Instead individual miRNAs are often used as controls (*e.g. miR-16, miR-24* and *miR-425* 10,19,126), however expression levels of these miRNAs can vary significantly among samples depending upon the pathology that is studied 127–129. We therefore propose that the least variable miRNA be determined empirically for each experimental cohort (using geNorm and/or NormFinder algorithms) 126,129. Alternatively, as sample volumes are often limited, we suggest that at least two endogenous short RNA controls should be used as standard in circulating transcriptome studies.

## Origin and function of cell-free nucleic acids

Although several non-exclusive hypotheses have been proposed to explain the source of circulating nucleic acids, their origin remains a contentious issue 130,131. Passive release of nucleic acids can occur from broken cells and tissues following tissue injury, chronic inflammation, cell apoptosis or necrosis, or also from cells with a short half-life, such as platelets 132–134 (Fig. 1). In addition, cfRNA can be actively secreted from cells; either in association with membrane-derived vesicles such as exosomes and microparticles 19,135–137, or alternatively conjugated with lipoproteins or RNA-binding proteins such as nucleophosmin 138, high-density lipoprotein 139 and Argonaute 1 and 2 (Ago1 and Ago2) 132,140. Until a couple of years ago, it was believed that the vast majority of circulating miRNAs were associated with membranous vesicles 135; however in 2011, two independent research groups reported that >90% of extracellular blood miRNAs were not present in vesicles but instead complexed with Ago proteins 132,140. However, more recent evidence suggests that at least some specific miRNAs in blood and saliva are present in higher quantities in vesicle fractions 141. Irrespective of origin, the composition of extracellular miRNAs differs from the profile of their respective donor cells 142, even to the point where secreted miRNAs are not observed in parental cells at all 137. This suggests that extracellular miRNA secretion is a highly specific phenomena and therefore likely to have biological significance.

A functional role for extracellular miRNAs was first demonstrated in plants in 1996 where they were shown to act as systemic signalling molecules 143, but it was over a decade later before this potential was first recognized in mammals 10,19. The ability of miRNAs to act as chemical communicators between cells, acting either in a hormone-like (endocrine) manner connecting disparate sites within the body and/or over short distances between cells as a paracrine signaller 131,144, has created much interest in recent years. Several factors support this general hypothesis, firstly that miRNAs are selectively packaged and secreted through highly regulated mechanisms 138,145. Secondly, extracellular miRNAs are protected from RNase activity in the blood by association with proteins and lipid carriers 140. Finally, that extracellular miRNAs are not only to be taken up by recipient cells but also able to alter their gene expression and mediate functional changes 137,142,146,147. The first example of this was the observation that exosomal miRNAs could be transferred between mast cells 137. Later on it was demonstrated that miRNAs could be transferred between embryonic stem cells and fibroblasts 148. More recently, exosomal miRNA released by T cells, B cells and dendritic cells were shown to be transferred to antigen-presenting cells modulating the gene expression of recipient cells 142. Multiple studies suggest that intercellular miRNA communication could play a role in cancer biology. For example, specific miRNA transport between IL-4-activated tumour-associated macrophages and breast cancer cells resulted in increased cell invasiveness 149, and the release of miRNA-containing vesicles from renal cancer stem cells stimulated both angiogenesis and metastasis 150,151. Furthermore, leukaemic cells were found to transfer *miR-92* exosomally to endothelial cells resulting in their increased cellular migration 152. It should be pointed out, however, that studies to date are almost exclusively *in vitro* and that the physiological relevance of extracellular RNA as an intercellular signalling mechanism remains to be determined, particularly as the concentration of extracellular RNA (∼100 fM) is much lower than even lowest trace hormone levels (∼1 pM) 153.

## Extracellular RNA of non-human origin: you are what you eat?

In 2012, Zhang *et al*. suggested that miRNAs derived from ingested plants could cross the gut–blood barrier and enter the blood stream, and that furthermore these miRNAs could regulate recipient human endothelial cells 154. Subsequently, a number of reports have supported this finding and there is increasing evidence from NGS data that plasma appears to contain a significant fraction (up to 40% 155) of non-human RNA originating from exogenous species including viruses, bacteria and fungi, as well as from common food species 154–157. However, some authors have challenged this data suggesting that contamination can account for most of these results 121. Nevertheless, this leads to the intriguing possibility that therapeutic ncRNAs could be administered to the population by incorporating them in food directly, or even that genetically modified crops could be engineered to express, for example, miRNAs (or antimiRs) with anti-cancer properties.

## Conclusion

The study of the circulating transcriptome continues to grow at a phenomenal rate and nowhere is this pace of discovery more rapid than their use as novel biomarkers of cancer. This ‘gold rush’ however, should be treated with some caution as the degree of discordancy between seemingly identical studies is worrisome, and in reality very few of the biomarkers studies published will ever make it into clinical practice. One notable exception to this is lncRNA PCA3, an FDA-approved biomarker (‘PROGENSA PCA3 test’) that has improved the specificity from 47% (PSA levels only) up to a 76% (PCA3 levels) for monitoring disease progression in prostate cancer patients whose PSA levels are elevated in serum after a negative biopsy detection 158.

Consequently, there is a clear need for a standardized approach to be taken in future cfRNA biomarker studies to rationalize these confounding factors. Another important factor to take into account is the lack of specificity of cfRNAs as biomarkers, illustrated by the fact that only a few cfRNAs are associated with patient survival in a wide variety of cancer types (Table 1).

In terms of future developments, apart from the need for more robust biomarker studies, which we might expect will be implemented and driven by commercial entities, an improvement in detection technologies and the development of dedicated biosensors would be the next logical step. Whatever happens, the future for cfRNA-based cancer biomarkers is very promising, and we should remember that we are only at the very beginning of our understanding of ncRNA and that in reality, miRNAs represent the tip of the ncRNA ‘iceberg’. Indeed, although ∼75% of the human genome is transcribed 159, the protein-encoding portion of the genome only accounts for 1.5% 160, while miRNAs represent another 1.8% 159 and it is surely only a matter of time before other classes of ncRNA are implicated as potentially useful circulating biomarkers.
